# Review of the development of DNA methylation as a marker of response to neoadjuvant therapy and outcomes in rectal cancer

**DOI:** 10.1186/s13148-015-0111-3

**Published:** 2015-07-22

**Authors:** Jeremy S. Williamson, Dean A. Harris, John Beynon, Gareth J.S. Jenkins

**Affiliations:** Department of Colorectal Surgery, Abertawe Bro Morgannwg University Local Health Board, Singleton Hospital, Sketty Lane, Swansea, SA2 8QA UK; Institute of Life Science, Swansea University, Singleton Park, Swansea, SA2 8PP UK

**Keywords:** Colorectal neoplasms, CpG island, Methylation, Neoadjuvant therapy

## Abstract

There is much debate around the preoperative treatment of colorectal cancer and, in particular, neoadjuvant chemoradiotherapy in locally advanced rectal cancer. This treatment carries a significant risk of harmful side effects and has a highly variable response rate. Predictive biomarkers have been the subject of a great deal of study with the aim of pretreatment risk stratification in order to more accurately determine which patients will derive the most benefit and least harm from these treatments. The study of epigenetics in colorectal cancer is relatively recent, and distinct patterns of aberrant DNA methylation, in particular the cytosine-phosphate-guanine (CpG) island methylator phenotype (CIMP), have been demonstrated in colorectal cancer, and their characterisation and significance are under debate, particularly in rectal cancer. These patterns of DNA methylation have been associated with differences in response to therapy and treatment outcomes and therefore have the potential to be used as biomarkers in tailored therapy regimes for patients with rectal cancer. This review aims to summarise the current state of the art in rectal cancer, with particular regard to the determination of DNA methylation patterns, the CpG island methylator phenotype and its potential as a novel biomarker in rectal cancer treatment and prediction of outcomes and response after neoadjuvant chemoradiotherapy.

## Introduction

Neoadjuvant chemoradiotherapy is currently recommended by the National Institute for Health and Clinical Excellence (NICE) [1] prior to surgical resection for patients with advanced stage (T3–4) rectal tumours and those with nodal disease in order to reduce rates of locoregional recurrence and potential circumferential margin involvement.

It is increasingly recognised that this preoperative chemoradiotherapy is associated with significant adverse effects including faecal and urinary incontinence, delayed wound healing and sexual dysfunction [2]. Presently, there are no biomarkers to predict the response of patients to this treatment, which in itself is highly variable. Outcomes vary from the favourable ‘pathological complete response’ which occurs in 10–20 % [3] of patients, with up to 30 % of patients who have no response to treatment [4].

If an accurate method to predict response to chemoradiotherapy was available, then patients with radiation-resistant disease would be spared the morbidity of this treatment, and those with a prediction of pathological complete response may ultimately be spared radical surgery.

Recently, attempts have been made to predict rectal cancer patient’s response to chemoradiotherapy including markers of cellular hypoxia [5], cellular expression of proteins such as *COX2* and *CD133* [6] and various protein kinases [7]. The role of *KRAS* and *BRAF* mutations has already been well established in their contribution to resistance to anti-EGFR agents [8], but no role in response to irradiation has been demonstrated [9, 10].

By contrast, studies of epigenetic silencing by aberrant DNA methylation in a number of cancers, including cervical [11], glioma [12], prostate [13], lung [14] and oesophageal cancers [15], have revealed novel insights into the response of tumours to radiotherapy with or without chemotherapy. There have been similar efforts in the study of DNA methylation in rectal cancers, and this review aims to summarise the current state of the art in this group of patients.

## Review

### DNA methylation and the CpG island methylator phenotype (CIMP)

Aberrant DNA methylation of cytosine-phosphate-guanine (CpG) islands has been reported widely in colorectal tumours and is associated with gene silencing when it occurs in promoter areas [16, 17]. CpG islands are typically short (300–3000 base pairs) cytosine-phosphodiester-guanine-bonded sequences found in or around the promoter region of a gene where they are usually unmethylated if the genes are expressed.

Methylation affects gene expression directly by interfering with transcription factor binding [18] and/or indirectly by recruiting histone deacetylases through methyl-DNA-binding proteins [19] to induce histone modification towards a more compacted and repressive chromatin state. In colorectal cancer cells, loss of DNA methylation has been demonstrated to induce a conformational change at histone H3K27 re-establishing its active transcriptional state resembling that of a methylation-deficient cell line [20].

The CpG island methylator phenotype (CIMP) is characterised by epigenetic DNA hypermethylation and consequent suppression of key groups of genes that are differentially methylated between normal and malignant cells and are important in controlling cell growth and survival.

DNA methylation is therefore necessary for the normal growth and development of cells and can be detected in normal as well as malignant colorectal mucosa [21]. In colorectal cancer, tumours with high-level CIMP-associated methylation (CIMP high) are associated with a particular subset of clinicopathological features including a predilection for mucinous tumours of the proximal colon and association with *BRAF* mutation [22]. CIMP-high tumours are associated with between 2 and 10 % of rectal cancers [23, 24]. However, some debate exists as to the existence of an intermediate group with a low level of CIMP-associated methylation (CIMP low) [25] which may also demonstrate distinct features, including a strong association with *KRAS* gene mutation [26].

### CIMP classifications and methods for determining methylation status in colorectal cancer

The classification of CIMP status has been under regular development since first reported by Toyota et al. [27]. This group used combined bisulphite restriction analysis (COBRA) to quantify DNA methylation in a set of 33 cancer-specific genes and found 7 of these were differentially methylated in a subset of cancers termed CIMP positive.

Alternative methods for methylation analysis utilise bisulphite conversion of DNA with subsequent direct Sanger sequencing or more accurate methods of quantification, such as pyrosequencing [28]. Methylation-specific PCR (MSP) is a further technique utilising bisulphite conversion although it avoids the use of restriction enzymes or sequencing and instead uses PCR primers that are designed to be specifically complementary to either methylated or unmethylated bisulphite-converted DNA at CpG sites of interest. The so-called classical panel of methylation markers, consisting of *CDKN2A* (*p16*), *MINT1*, *MINT2*, *MINT31* and *MLH1*, was developed by Park et al. [29] using MSP, and methylation of two or more markers in this panel was deemed CIMP high (CIMP H). Limitations of MSP include false positive results caused by incomplete bisulphite conversion and denaturation of DNA caused by the bisulphite itself. Furthermore, it only provides a qualitative result, and although still popular due to its relative low cost and high throughput, other more quantitative techniques such as high-resolution mass spectrometry may provide a more accurate characterisation of methylation status [30].

The MethyLight assay developed by Eads et al. [31] utilises a refinement of MSP and has been used to quantitatively assess methylation and is designed for high throughput, small amounts of DNA and analysis of multiple gene loci without the need for gel electrophoresis. DNA is bisulphate converted, amplified by reverse transcriptase quantitative PCR using primers containing an oligonucleotide probe with fluorescent dye reporter. The intensity of fluorescence is proportional to methylation quantity.

MethyLight analysis was used by Weisenberger et al. [16] to further strengthen the evidence for a subset of CIMP colorectal cancers. This group used MethyLight analysis and hierarchical clustering to confirm a CIMP-positive subset of cancers which was strongly related to mutation of the *BRAF* gene [16]. This analysis used five methylation-associated gene promoters, the so-called alternative panel including *CACNA1G*, *IGF2*, *NEUROG1*, *RUNX3* and *SOCS1*, and classified tumours as CIMP positive if four or more than five of this panel of promoters were methylated.

Further investigation with this technique by Ogino et al. [32] who examined methylation in 840 colorectal cancers led to the proposal that a further subset of methylation-associated tumours exists but which does not fulfil the criteria for CIMP high. These tumours (termed CIMP low if 1-3/5 promoters were methylated) were independently associated with male gender and *KRAS* mutation. The three-epigenotype model was further supported by Yagi et al. [25], who used a large-scale mass spectrometry analysis and hierarchical clustering to identify two panels of markers, the first to identify CIMP-high tumours and then a second panel to distinguish between CIMP-intermediate and CIMP-low tumours. This group has also extended the CIMP-intermediate *KRAS* association to colorectal adenomas [33]. There is as yet no consensus on the definitive panel for classifying CIMP status; indeed, a recent study by Berg [34] comparing several CIMP panels found significant variability between published panels, with a twofold difference in CIMP positivity determined by the most and least stringent panels.

In contrast to examining methylation at a small number of loci, more recently, investigators have adopted a genome-wide approach to the analysis of DNA methylation, extending the investigation to beyond the CIMP classification. Beggs et al. [35] used a methylation analysis based on bisulphite conversion and whole-genome amplification and found that in ten colorectal cancer and five colorectal adenoma specimens, quantification of whole-genome methylation did not correlate with previously published CIMP criterion. They also noted increasing levels of methylation from progression of normal tissue through adenoma and cancer cells.

Differential methylation across various individual and groups of genes has therefore demonstrated distinct associations with various clinicopathological features of colorectal cancer. It is therefore possible that these associations may extend to differential response rates to neoadjuvant treatment in rectal cancers, and the current state of the art is discussed below.

### Cell line studies

A small number of studies have modelled the effect of radiotherapy upon colorectal cancer cells and the role of differential methylation in determining the response of these cells.

Kim et al. [36] investigated the role of methylation of the ataxia telangiectasia mutated (*ATM*) gene in response to 10 Gy of ionising radiation (IR) in mismatch-repair-deficient cell lines from patients with hereditary non-polyposis colorectal cancer. They found a significantly greater response to IR in cells with a hypermethylated *ATM* gene promoter that was found to be associated with reduced ATM expression, and this was reversed by treatment of cell lines with 5-azacytidine (5-AZA) a demethylating agent. Similar results have been demonstrated in glioma cells, whereby ATM promoter methylation was associated with twofold enhancement of radiosensitivity [37]. The *ATM* gene product is a protein kinase, activated by DNA damage, and initiates cell cycle arrest whereby either DNA damage repair or apoptosis occurs. Loss of this functioning gene is demonstrated in patients with ataxia telangiectasia who display 70 times higher risk of leukaemia and 250 times higher risk of lymphoma than normal individuals [38]. The hypermethylation-associated loss of *ATM* function thereby leads to gross genetic instability which may explain the enhanced radiosensitivity in these cells and should therefore be further investigated in studies of patients with rectal cancer.

In contrast, Hofstetter [39] reported that treatment of four CRC cell lines with AZA caused demethylation of *CDKN2A* (*p16*) and *hMLH-1* genes and resulted in enhanced radiation sensitivity of these cell lines treated with up to 10 Gy. The *p16* protein is a cyclin-dependent kinase inhibitor which regulates cell cycle progression, and *hMLH-1* is a DNA mismatch repair gene. Although a mechanism explaining this enhanced sensitivity with demethylation has not been elucidated experimentally, the role of these genes is closely linked at the G1/S transition phase of the cell cycle, and the subsequent fate of irradiated cells containing DNA damage at this point of the cell cycle would be influenced by alterations in expression of these two genes. In particular, one study which found enhanced radiosensitivity of CDKN2A mutant head and neck cancer cell lines found an extended G2 arrest phase and increased frequency of double-stranded DNA breaks in these cells and concluded that compromised DNA repair was responsible for this enhancement of the response to radiotherapy [40].

These studies suggest that the role of methylation in response to radiation may be specific to several factors including genotype and target tissue, and although the effect of methylation status on radiosensitivity is as yet not clearly defined in colorectal cancer cell lines, studies in non-colorectal tissues have suggested that, overall, the least methylated tissues are more radiosensitive [41] and that the *de novo* DNA methyltransferases are important targets for future investigation for mechanistic explanations of radiosensitivity [42]. In clinical practice, a combination of radiotherapy with chemotherapeutic agents is used. The chemotherapeutic agent 5-fluorouracil in combination with radiotherapy is the most well-established neoadjuvant regime [43] and has been demonstrated to increase the sensitivity of cells to radiotherapy, thereby improving the local effectiveness of radiotherapy *in vitro* and in patient studies [44]. The relative combination of chemotherapy, radiotherapy and methylation status to outcome has not been well investigated at the cellular level, presumably due to the technical difficulties in creating a representative cell line model.

### Tumour regression in response to neoadjuvant treatment and relationship to methylation

There has also been relatively little *in vivo* or *ex vivo* study of the role of methylation in determining prognosis or response of rectal cancer to treatment.

Few studies have investigated the role of methylation with specific regard to its effect upon tumour regression or response to neoadjuvant chemoradiotherapy. Ebert et al. [45] reported a significant relationship between hypermethylation of the transcription factor activating protein 2 epsilon (*TFAP2E*) gene and response to neoadjuvant chemoradiotherapy. They found that patients with either primary rectal cancer or metastatic colorectal cancer with *TFAP2E* gene hypermethylation showed subsequent reduction in *TFAP2E* protein expression and poor response to treatment as assessed both histologically and by radiological criteria. With specific regard to the response of rectal cancer patients, only 10 % of patients with hypermethylated *TFAP2E* showed a tumour regression response in comparison to 82 % of those with hypomethylated *TFAP2E*. These differences were seen in patients treated with neoadjuvant radiotherapy and the chemotherapeutic agent 5-fluorouracil (5-FU), either alone or in combination with irinotecan and cetuximab. The authors suggested *TFAP2E* downregulates *DKK4*, itself an antagonist of the wingless/integration (WNT) signalling pathway. This pathway has also been implicated in hypermethylation-mediated effects on the response of other cancers including head and neck and ovarian [11] although the precise mechanisms mediating radioresistance are still unknown. Recently, Beggs et al. have identified TFAP2E hypermethylation to actually confer a survival advantage in colorectal cancer subsets and have suggested differential methylation of TFAP2E may be more complex than previously understood. They identified differential hyper- and hypomethylation across various CpG sites within the TFAP2E promoter region and noted, unlike Ebert, a significant relationship between this and BRAF mutation, suggesting that the MAPK pathway may also mediate the relationship between TFAP2E methylation and outcomes [46].

In the same year, Jo et al. [23] examined the CIMP status (classifying into either positive or negative) in over 100 patients with rectal cancer undergoing 5-FU-based neoadjuvant chemoradiotherapy. Post-chemoradiotherapy tumour regression in the resected specimen was compared with the pretreatment biopsy CIMP status; however, no significant relationship between these variables was found. Also, in contrast to other investigators, no association was reported between CIMP status and *KRAS* or *BRAF* mutation.

In contrast to the above group, recently, Sun et al. [47] examined the response to 5-FU-based chemoradiotherapy in 219 rectal cancer patients and found improved response to treatment in those patients with hypermethylated *MGMT* promoters at baseline. In this study, 88.9 % of patients with good response and higher regression scores demonstrated hypermethylation of MGMT, compared to 50 % of those in the poor response group (*P* = 0.04).

Although a precise mechanism has not been identified in the literature, *MGMT* is a DNA repair enzyme and it is therefore logical that hypermethylation and subsequent inactivation of this enzyme could lead to an enhanced effectiveness of ionising radiation by reducing the cells’ capacity to repair the nuclear damage inflicted by the therapy, thereby driving the cell into apoptosis.

Similarly, Molinari et al. [48] found hypermethylation of the *TIMP3* tumour suppressor gene appeared to be associated with improved response to 5-FU-based neoadjuvant chemoradiotherapy. They found that tumours demonstrating no response to CRT harboured significantly lower rates of TIMP3 methylation compared to those demonstrating some response (1–2 methylation frequency 1–2 % vs 2–3 %, *P* = 0.015) although the association did not reach statistical significance for comparison of complete responders vs non-responders, and there was wide variation in the data. Furthermore, examination of 23 other tumour suppressor genes in the same analysis yielded no differences in methylation and response; therefore, the authors urged caution that *TIMP3* is not likely to be a principle marker of radiosensitivity in rectal cancer.

This science is still in its infancy, and as yet, there have been no large-scale studies investigating multiple panels of markers of methylation and their specific role in radiosensitivity of rectal cancer. To date, the best available evidence is from heterogenous studies investigating individual or small groups of relatively unrelated genes, and as such, it is difficult to draw wider ranging conclusions. From the limited published studies, it is clear that the relationship between response and methylation can be contrasting and depends upon the target gene studied, and although some promise has been demonstrated in a few markers, larger prospective studies are required before these could impact clinical practice.

### Outcomes and survival in relation to methylation

There are conflicting reports in the literature regarding the prognostic implication of CIMP status and DNA methylation as a whole.

Several studies have implicated CIMP positivity as an adverse survival predictor in patients with colorectal cancer [28, 49, 50]; however, the majority of studies investigating survival outcomes in relation to methylation status regard colon and rectal cancers as one entity.

A study of rectal cancer patients by Jo et al. [23], also discussed in the previous section, also compared survival after neoadjuvant chemoradiotherapy in over 100 rectal cancer patients. The 3- and 5-year survivals were significantly worse in the 10 % of patients who were CIMP positive (56 and 0 % vs 80 and 75 %; *P* < 0.01).

These findings, however, were not corroborated by Kohonen-Corish et al. [51] who, amongst other molecular features, examined CIMP status and *CDKN2A* gene methylation in nearly 400 rectal cancers. They found CIMP-H status was a rare phenomenon (4 %), but was not itself associated with adverse outcomes including survival.

*CDKN2A* methylation itself was again not associated with poor survival; however, the joint presence of *CDKN2A* methylation and *KRAS* mutation was independently associated with poor survival and increased risk of local recurrence. In this study, 95 % of patients had not received neoadjuvant chemoradiotherapy thereby making it difficult to determine the relative contribution of this treatment when making comparisons with other studies.

*CDKN2A/p16* methylation has been the subject of several studies, and two recent meta-analyses including over 3000 patients with colorectal cancer have revealed a strong association of *CDKN2A/p16* methylation with adverse survival, lymph node metastasis and lymphovascular invasion [52, 53].

Bae et al. [54] reported worse survival in CIMP-H colorectal cancer patients; however, after multivariate analysis, only CIMP-H rectal cancers (*n* = 168) remained significantly associated with poor survival (HR 4.13, *P* = 0.019) in contrast to other colorectal subsites which were not significant. Patients undergoing neoadjuvant chemoradiotherapy were excluded in this study.

Conversely, genomic hypomethylation has been reported as a poor prognostic feature in several studies.

Benard et al. [55] performed a similar analysis for nearly 100 patients with early rectal cancer and found that hypomethylation of the long interspersed nuclear element type 1, L1 (*LINE-1*) non-coding DNA repeat sequence was associated with worse survival (hazard ratio 4.56, *P* = 0.014) and higher risk of tumour recurrence (hazard ratio 9.57, *P* = 0.001). As *LINE-1* repeat sequences form approximately 17 % of the human genome, these were considered to be a marker of global methylation in this study. Similarly, these patients had not undergone neoadjuvant radiotherapy, making it difficult to determine the role of this marker in the effect of response to neoadjuvant treatment. LINE-1 sequences are capable of their own expansion and mobilisation and contribute to genetic recombination events that can dramatically alter the shape of the genome. LINE-1 activation may result in genetic deletions and double-stranded DNA breaks which contribute to genetic instability. Studies in rats [56] have demonstrated ionising radiation caused a reduction in LINE-1 promoter methylation, increasing its activity, which may contribute to genetic instability and provide a mechanism for the phenomena observed by Benard.

Recently, Gaedcke et al. [57] applied a whole-genome methylation analysis to identify several differentially methylated regions (DMR), in which high-pretreatment levels of methylation imparted a better prognosis in terms of disease-free survival in 165 patients in three cohorts treated for locally advanced rectal cancer (hazard ratios between 3.57 and 4.09, *P* < 0.05). These cohorts included a tight group of selected patients undergoing standardised 5-FU-based chemoradiotherapy as well as a more heterogenous group undergoing radiotherapy as monotherapy or in combination at various doses with various agents including oxaliplatin. However, this study did not a report specific analysis of tumour regression. Interestingly, the authors applied a similar analysis to previously reported CIMP markers (which did not feature in the main DMR study group) and found some minor differences in disease-free survival between CIMP groups, with the hypermethylated phenotype imparting a better survival, although this did not reach significance and did not improve the prognostic utility when combined with the DMR panel proposed by this study.

Simultaneous hyper- and hypomethylation within patients has also been demonstrated to have some prognostic utility. De Maat et al. [58] investigated methylation at several methylated in tumour (MINT) loci. These MINT loci are non-protein-encoding CpG-rich regions which have been previously identified as hypermethylated in colorectal cancer and form part of many CIMP marker panels. This group identified a cohort of rectal cancer patients undergoing surgery without neoadjuvant treatment in whom hypermethylation at MINT3 and hypomethylation at MINT17 loci were associated with a significantly reduced risk of local recurrence compared with others not demonstrating these epigenetic features. This group (which may account for up to 30 % of all rectal cancers) was also compared to patients undergoing neoadjuvant chemoradiotherapy and surgery and was found to have similar local recurrence rates after 8 years of follow-up, indicating that this group may be spared the risks of neoadjuvant chemoradiotherapy with no significant increase in recurrence rates. The MINT loci have no protein-encoding function of their own; however, they lie near coding regions for genes including *RBBP4*, *YARS* and *HRK* which are involved in histone deacetylation, angiogenesis and apoptotic regulation, respectively. No functional or regulatory relationship has been identified between MINT3 and MINT17, and so, the authors conclude their role is of a surrogate biomarker at present.

It is likely that the heterogeneous nature of colorectal cancer patients, CIMP marker panels, tumour location along colorectal subsites and the highly complex nature of the multitude of molecular pathways and their interactions is only just being realised, and more work will be required to clearly elucidate these complex interactions. Indeed, there is emerging evidence that given their separate embryological origins, the rectum should be considered as a separate organ to the colon [59] and that this is manifest not least by differences in methylation status along the length of the lower GI tract [24]. Heterogeneity of DNA and epigenetic phenomena has also been implicated within tumours and subsequent sampled cell types [60] and across patient age groups [61]. It is likely that variability between these features as well as gender and race may also account for differences observed in the prognostic utility of methylation markers across various studies.

Most investigators identify CIMP as an adverse prognostic feature, particularly in colorectal cancer taken as a whole, and this was also corroborated by a recent meta-analysis including all colorectal subsites, which found shorter survival in CIMP-positive patients [62]. The authors of this analysis stated that the limitations of their analysis are due to the lack of a standardised characterisation of CIMP status. Furthermore, no mechanism for the role of CIMP in adverse prognosis of colorectal cancer has yet been proposed and satisfactorily demonstrated in the literature. See Table 1 for summary of methylation markers discussed.

**Table 1:** Summary of methylation markers

Author	Marker	Study population	Response to treatment	Treatment given	Other findings
Kim 2002 [36]	ATM methylation	Human HNPCC-deficient CRC cell lines	Hypermethylation associated with increased response to IR	10 Gy radiation	Reversal of gene suppression and increased response after AZA treatment
Hofstetter 2010 [39]	P16 and hMLH-1 methylation	Human CRC cell lines (×4)	Demethylation of markers resulted in enhanced radiation sensitivity	10 Gy radiation	N/A
Giotopoulos 2006 [41]	Quantitative DNA 5-methyl-cytosine content analysis	Murine bone marrow cells	Hypomethylation of bone marrow associated with increased radiation sensitivity	3 Gy radiation	N/A
Ebert 2012 [45]	TFAP2E methylation	110 human locally advanced rectal cancers	Hypermethylation associated with markedly reduced response to neoadjuvant CRT (10 vs 82 %)	Treatment dose radiotherapy with combination chemotherapeutic agents	Possible mechanism via WNT signalling pathway
Jo 2012 [23]	CIMP status +/−	150 human locally advanced rectal cancers	No association of pathological response to IR with CIMP status	Treatment dose radiotherapy and 5-FU chemotherapy	Increased risk of distant metastases and poorer 5-year survival with CIMP +
Sun 2014 [47]	MGMT methylation	219 rectal cancer patients	MGMT hypermethylation associated with increased tumour regression	Treatment dose radiotherapy and 5-FU chemotherapy	N/A
Molinari 2013 [48]	TIMP3 methylation	74 rectal cancer patients	TIMP3 hypomethylation associated with poor tumour regression	Treatment dose radiotherapy and 5-FU chemotherapy	Several genes including APC found differentially methylated between tumour and normal mucosa
Kohonen-Corish 2013 [51]	CIMP H/L and CDKN2A methylation	381 early rectal cancers	Not examined	Primary surgery for majority of patients	CIMP H or CDKN2A not independently associated with survival. CDKN2A and KRAS mutation associated with poor survival and increased recurrence
Bae 2013 [54]	CIMP status H/L/0	168 rectal cancers (stages I–IV)	Not examined	Primary surgery only	CIMP H associated with poor survival
Benard 2013 [55]	LINE-1 methylation	94 early rectal cancers	Not examined	Primary surgery only	Hypomethylation of LINE-1 associated with increased risk of recurrence and poor survival
Gaedcke 2014 [57]	Whole-genome methylation	165 locally advanced rectal cancer patients	Not examined	3 cohorts including neoadjuvant 5-FU, oxaliplatin and radiotherapy	10 differentially methylated regions (DMRs), hypermethylation of which predicts improved disease-free survival
De Maat 2010 [58]	MINT loci	251 rectal cancer patients (stages I–III)	Not examined	Primary surgery only	MINT 3 hyper- and MINT 17 hypomethylation predicts reduced risk of recurrence similar to unselected patients undergoing neoadjuvant treatment

## Conclusions

The use of appropriate neoadjuvant treatment and surgical approach relies on accurate pretreatment risk stratification, particularly with regard to local invasion and circumferential margin involvement. The current gold standard staging tool is magnetic resonance imaging (MRI) which can be used to accurately predict those with ‘good prognosis’ tumours which may be managed with surgery alone resulting in local recurrence rates of just 3 % [63].

With these advances in risk stratification and surgical technique, there have been calls for a move away from the use of neoadjuvant chemoradiotherapy in selected cases [64]. The contribution of an effective and reliable biomarker to this risk stratification process is an exciting prospect, and there is evidence that methylation markers may have an important role. This may be used to identify likely non-responders in selected groups such as in borderline cases where there may be only marginal benefits of neoadjuvant treatment or those in which it is difficult to balance benefit against the risk of adverse effects, including disease progression where a delay in surgery could be detrimental. Currently, there are no means to predict response to treatment; however, methylation markers could be validated to identify those unlikely to respond to permit primary surgery. Conversely, if methylation markers are able to identify those likely to exhibit a pathological complete response, it is conceivable that patients could potentially be treated with neoadjuvant chemoradiotherapy.

Currently, the methods for determining methylation, classification into prognostic groups such as CIMP and their utility in predicting response to therapy remain under investigation. However, to date, the evidence suggests that both hyper- and hypomethylation may predict outcomes and response to treatment and that demethylation of tissues may improve response to ionising radiation. Furthermore, this evidence has been gathered from *in vitro*, *ex vivo* and *in vivo* studies. The role of combination biomarkers has not been well established, and this may require further clarification of the role of methylation in specific sites as well as collaborative research with other well-established predictive markers such as KRAS mutation which has already been associated with CIMP status. Our understanding of this area may be improved by refinement of the markers of the CIMP panel as well as identification of other prognostic associations of the CIMP phenotype. Furthermore, it is recommended that future studies report the behaviour of rectal and colonic cancers separately as it is evident that these are distinct entities, both in terms of response to treatment and molecular characteristics. Several potential methylation markers with clinical application have been identified in the literature; however, the mechanisms for their role are not yet well understood. In particular, the precise interactions between hyper- and hypomethylation of candidate markers are not clear, and it is proposed that further study in this regard is desirable. Furthermore, given the evidence for the predictive role of several key markers in determining response and outcomes, in particular TFAP2E, MINT and DMRs, further prospective trials of their use should be considered.
